# Changing Behavioral Lifestyle Risk Factors Related to Cognitive Decline in Later Life Using a Self-Motivated eHealth Intervention in Dutch Adults

**DOI:** 10.2196/jmir.5269

**Published:** 2016-06-17

**Authors:** Teun Aalbers, Li Qin, Maria AE Baars, Annet de Lange, Roy PC Kessels, Marcel GM Olde Rikkert

**Affiliations:** ^1^ Radboud University Medical Center Department of Geriatric Medicine Nijmegen Netherlands; ^2^ Radboud University Medical Center Radboud Alzheimer Center Nijmegen Netherlands; ^3^ HAN University of Applied Sciences Institute of Social Sciences Nijmegen Netherlands; ^4^ HAN University of Applied Sciences Faculty of Human Resource Management Nijmegen Netherlands; ^5^ Norwegian School of Hotel Management University of Stavanger Stavanger Norway; ^6^ Radboud University Nijmegen Donders Institute for Brain, Cognition and Behaviour Nijmegen Netherlands; ^7^ Radboud University Medical Center Department of Medical Psychology Nijmegen Netherlands

**Keywords:** lifestyle, risk reduction behavior, cognition, video games, telemedicine

## Abstract

**Background:**

Our labor force is aging, but aged workers are not yet coached on how to stay cognitively fit for the job.

**Objective:**

In this study, we tested whether a self-motivated, complex eHealth intervention could improve multiple health-related behaviors that are associated with cognitive aging among working Dutch adults.

**Methods:**

This quasi-experimental prospective study with a pre-post design was conducted with employees of Dutch medium to large companies. All employees with Internet access, a good understanding of the Dutch language, and who provided digital informed consent were eligible to participate. In total, 2972 participants (2110/2972, 71.11% females) with a mean (standard deviation, SD) age of 51.8 (SD 12.9) years were recruited; 2305 became active users of the intervention, and 173 completed the 1-year follow-up. This self-motivated eHealth lifestyle intervention stimulates participants to set personally relevant, monthly health behavior change goals using Goal Attainment Scaling and to realize these goals by implementing behavior change techniques grounded in behavior change theory. The primary outcomes were the goal-setting success rate and the change in overall lifestyle score from baseline to the 1-year follow-up; the score was based on physical activity, diet, smoking, alcohol, sleep, and stress scores. The secondary outcomes were the changes in body weight, body mass index, specific lifestyle characteristics, and website usage.

**Results:**

A total of 1212 participants set 2620 behavior change goals; 392 participants assessed 1089 (1089/2288, 47.59%) goals and successfully achieved 422 (422/1089, 38.75%) of these goals. Among the goal-setting participants in follow-up, this led to a +0.81-point improvement (95% CI 0.49-1.13, *P*<.001) in overall lifestyle (*d*=0.32) and weight loss of 0.62 kg (95% CI −1.16 to −0.07, *P*=.03). These participants also showed significant improvement in 8 out of 11 specific lifestyle components.

**Conclusions:**

Among an adult Dutch population, this eHealth intervention resulted in lifestyle changes in behavioral risk factors associated with cognitive decline, and these improvements lasted over the period of 1 year. Given the general aging of our workforce, this eHealth intervention opens new avenues for the widespread use of cost-effective self-motivated prevention programs aimed at prevention of early-stage cognitive decline and more self-management of their risk factors.

**Trial Registration:**

Nederlands Trial Register: NTR4144; http://www.trialregister.nl/trialreg/admin/rctview.asp?TC=4144 (Archived by WebCite at http://www.webcitation.org/6cZzwZSg3).

## Introduction

A number of large-scale longitudinal studies have shown that several behavioral risk factors are associated with the onset and progression of diabetes, cardiovascular disease, stroke, and cognitive impairment [1-3]. Moreover, studies have shown that the relative risk of developing these diseases increases when several risk factors are present [4]. Consequently, reversing unhealthy behaviors may significantly benefit one’s health in later life. Three key unhealthy behaviors—a lack of physical activity, consuming an unhealthy diet, and tobacco use—are estimated to account for approximately 71% of the more than 1 million preventable deaths that occurred in the year 2000 in the United States alone [5,6]. Moreover, a growing body of evidence suggests that good health during one’s midlife years has a positive effect on cognitive aging, and focusing on modifiable lifestyle-related risk factors can delay or even prevent the onset of Alzheimer disease [7-9]. Physical activity, nutrition, adiposity, smoking, alcohol consumption, sleep, and stress have all been identified as modifiable lifestyle factors that are associated with cognitive aging [10-18]. A recent study even predicted that one-third of all global cases of Alzheimer disease can be attributed to potentially modifiable risk factors [19], and according to a study by Barnes and Yaffe [7], even a 10%-25% reduction in modifiable risk factors for Alzheimer disease might prevent up to 3 million cases of Alzheimer disease worldwide.

### Internet-Based Lifestyle Programs

In a recent systematic review, we found that tailored Internet-based intervention programs provide an evidence-based means to effect large-scale change in modifiable risk factors [20]. The efficacy and feasibility of Web-delivered intervention programs for changing unhealthy behaviors is well established with respect to the relevant health behaviors (eg, increased physical activity, weight loss, smoking cessation, and reduced alcohol consumption) [21,22]. Moreover, more recent research attempted to determine which types of interventions are the most effective. For example, recent meta-analyses revealed that some form of tailoring is necessary for improving the personal relevance and extent of health behavior change programs [23] and for increasing the intervention’s effectiveness [24]. However, to date, no published eHealth study has been designed to investigate the effect of lifestyle changes on cognitive aging.

### Objective of the Study

Our objective was to design an innovative eHealth intervention program that motivates aging adults to adopt healthy lifestyle changes in order to help prevent cognitive decline. To facilitate feedback and to increase participant motivation, the intervention enables participants to monitor their own cognitive functioning over time by playing applied games (abbreviated here as the BAM-COG, Brain Aging Monitor–Cognitive Assessment Battery, part of the intervention), which provide a valid measure of various cognitive functions [25]. These games are part of the Brain Aging Monitor (BAM), an eHealth system with minimal barriers for participation, and they are sustainable in a wide range of practice settings. After developing and pilot-testing this eHealth intervention program, we initiated this study by asking whether using this self-motivated, complex eHealth intervention could effectively result in changing multiple health behaviors at midlife that are associated with cognitive decline in later life.

## Methods

### Study Design

This study used a pre-post design, in which newly enrolled participants chose their own time path, from registration to setting goals and monitoring their change in behavior. Randomizing the participants against a sham intervention in an occupational health promotion program, in which the stimulated behaviors are known to be advantageous, was judged to be insufficiently motivating for the participants and potential participating companies; therefore, we chose to use a pre-post design. Inclusion started in October 2012. This intervention was registered with the Dutch Trial Register (NTR4144) and was exempt from formal testing by the Medical Ethics Committee of the Radboud university medical center Nijmegen, which determined that the intervention was not invasive, risky, or burdensome.

### Study Population

Participants were recruited from medium to large companies and from the general population. Due to the Internet-based nature of the eHealth intervention, no regional restrictions applied. Although the intervention was primarily aimed at participants aged 40 years and older, no age restriction for registration applied, as this did not result in any additional logistics or cost. Participants were required to have regular Internet access at their work and/or home. This was not a relevant barrier to participation, as approximately 92% of individuals aged 45-75 years in the Netherlands have Internet access [26]. Because the intervention was available only in Dutch, a good understanding of the Dutch language was a prerequisite for registration, and all participants were required to provide electronic written informed consent. Because the presented outcomes are intermediate outcomes for the overall 2-year follow-up data outcomes, no power analysis was performed specifically for the reported outcomes.

### Assessment of Risk Factors Related to Cognitive Aging

At baseline, physical activity, nutrition, smoking, alcohol consumption, sleep patterns, and stress behavior data were collected using electronic questionnaires. For a detailed overview of the room for improvement in these 6 lifestyle factors in Dutch society, see Multimedia Appendix 1: Room for improvement in the Netherlands. We monitored the participants’ cognitive functions (eg, working memory, visuospatial short-term memory, and planning performance) using the BAM-COG, an Internet-based tool for self-monitoring cognitive functioning using applied games that we previously developed and validated [25]. In addition, we administered the Dutch General Self-Efficacy Scale [27], lifestyle factor–specific self-efficacy questions [28], the Positive and Negative Affect Schedule [29], and the Self-Control Scale [30]. A complete overview of these questionnaires is available from the protocol [31]. Twelve months after starting the intervention, the participants were automatically prompted by email to repeat the e-questionnaires and BAM-COG. We monitored the number of log-in events, the number of goals set, the number of goals assessed (ie, the goals that were scored by the participant), and whether or not a goal was achieved. Goals were set using Goal Attainment Scaling (GAS; see the example in Table 1; number of days and length of exercise bouts are variable) [32]. The main features and strengths of using GAS are that it enables BAM to compare goals over different lifestyle modalities, it provides positive feedback regarding partially accomplished goals, and it stimulates the participant to consciously consider what goals are realistic within a given time frame.

**Table 1:** One possible example of a filled out Goal Attainment Scaling for the goal “I want to exercise more.”

	Behavior frequency		Behavior duration	GAS^a^ score
I fall short of my goal if I exercise	1 time per week	For	45 minutes	−2
I fall a little short of my goal if I exercise	2 times per week	For	45 minutes	−1
I reach my goal if I exercise	3 times per week	For	45 minutes	0
I exceed my goal if I exercise	4 times per week	For	45 minutes	+1
I greatly exceed my goal if I exercise	5 times per week	For	45 minutes	+2

^a^ GAS: Goal Attainment Scaling.

Using these GAS scores, we measured both overall success and the success of each specific lifestyle area (as the percentage of achieved goals out of the total number of goals set). To measure overall lifestyle change, we calculated an overall lifestyle score based on the following 8 lifestyle measures: physical activity, exercise, healthy nutritional behavior, unhealthy nutritional behavior, smoking status, alcohol consumption, sleep status, and stress status. Each of these factors was categorical with a value of 1-3, thus summing to a total score that ranged from 8 (ie, an unhealthy lifestyle) to 24 (ie, a healthy lifestyle). More detailed information regarding how the values were defined for each lifestyle factor is provided in Multimedia Appendix 2: Construction of the overall lifestyle score. It is important to note that an outcome on a GAS score does not directly match a change in category in Multimedia Appendix 2. They are, however, related in such a way that if the participant in the example of Table 1 would obtain a GAS score of 0 or higher, he or she would switch from the category of “suboptimally active” to “norm active.”

### The Brain Aging Monitor Intervention

Participants were able to register free of charge at the intervention website. After providing electronic written informed consent and receiving email validation of their account, the participant could log on to a personalized “dashboard.” After completing the questionnaires (for their tailored intervention content), each participant received a personalized lifestyle overview indicating room for improvement, after which the participant was invited to complete the 3 validated Internet-based puzzle games to assess their baseline cognitive performance.

After the questionnaires were completed, the intervention components were unlocked, thus enabling the participant to begin setting behavioral goals using the GAS methodology [32] in the BAM interface. After a participant set a goal, positive reinforcement was provided, along with practical tips and tricks to accomplish that specific goal (eg, when a participant’s goal was to “start exercising,” the tips and tricks provided included training schedules building up to a 5-km run or 500-m swim). Because using behavior change techniques that are based on evidence-based principles leads to better final scores [33], we incorporated 13 of the 26 behavior change techniques that were identified in the taxonomy by Abraham and Michie [34]. These techniques are grounded in the Social Cognitive Theory [35,36], the Transtheoretical Model [37], the Theory of Reasoned Action, and the Theory of Planned Behavior [38]. Each goal was then transferred to the short-term monitoring system, in which the participants could monitor their behavior on a daily basis; behavior was represented graphically in bar charts. After 1 month, the participants were asked whether the goal was achieved. If the participant answered this question (regardless of the answer), the goal was considered assessed. If the result was positive (ie, a GAS score of ≥0), the goal was registered as being achieved successfully and the goal was transferred to the long-term monitoring system (LTMS); if the result was negative (ie, a GAS score of <0), the goal was deleted from the participant’s profile. After a goal with a positive outcome was entered into the LTMS, it was monitored monthly in order to track the behavior and stimulate its maintenance.

The BAM automatically sent reminder emails to the participants each week. The frequency of these reminder emails could be changed by the participant to daily, biweekly, or monthly intervals. In addition to the goal-setting and monitoring components, the BAM also featured weekly blogs and healthy recipes [31].

### Primary and Secondary Outcomes

We defined changes in risk factors for cognitive aging and website use as the 1-year outcomes. The cognitive outcome measures require longer follow-up (eg, 2 years) and were not available at the time of publication [31]. The first 1-year primary outcome was the overall and lifestyle-specific goal-setting success rates, which were calculated as GAS scores of ≥0. The second primary outcome was the change in overall lifestyle score from baseline to the 1-year follow-up. The secondary outcomes were changes in body weight, body mass index (BMI), and the resulting changes in specific lifestyle areas.

### Data Analyses

The differences between the group of participants who set goals after doing the pretest (goal-setting group) and the group of participants who did not set goals after the pretest (non–goal-setting group) at baseline were analyzed using the independent samples *t* test (for interval variables) or the χ^2^ test (for categorical variables). The Mann-Whitney *U* test was used to compare differences in intervention usage. We used the paired samples *t* test to analyze within-group differences in overall lifestyle score, BMI, weight, and lifestyle-specific areas. An analysis of covariance (ANCOVA) was used to calculate the mean change in body weight, BMI, and lifestyle changes, as well as the 95% CI. For changes in body weight and BMI, the baseline values were adjusted as covariates in the ANCOVA in order to control for a potential regression-to-the-mean effect. After adjusting for covariates, multivariate linear regression analyses were performed to assess the linear associations between the total number of goals set (as a proxy for intervention utilization) and the changes in lifestyle factors at the 1-year follow-up. Unless stated otherwise, the outcome values are presented as the mean and standard deviation (SD); where possible, 95% CI is presented as well. Effect size (Cohen’s *d*) was calculated for the overall lifestyle change. All *P* values are based on 2-sided testing, and all statistical analyses were performed using SPSS version 20 (SPSS Inc, Chicago, IL, USA).

## Results

### Baseline Characteristics

**Figure 1:**
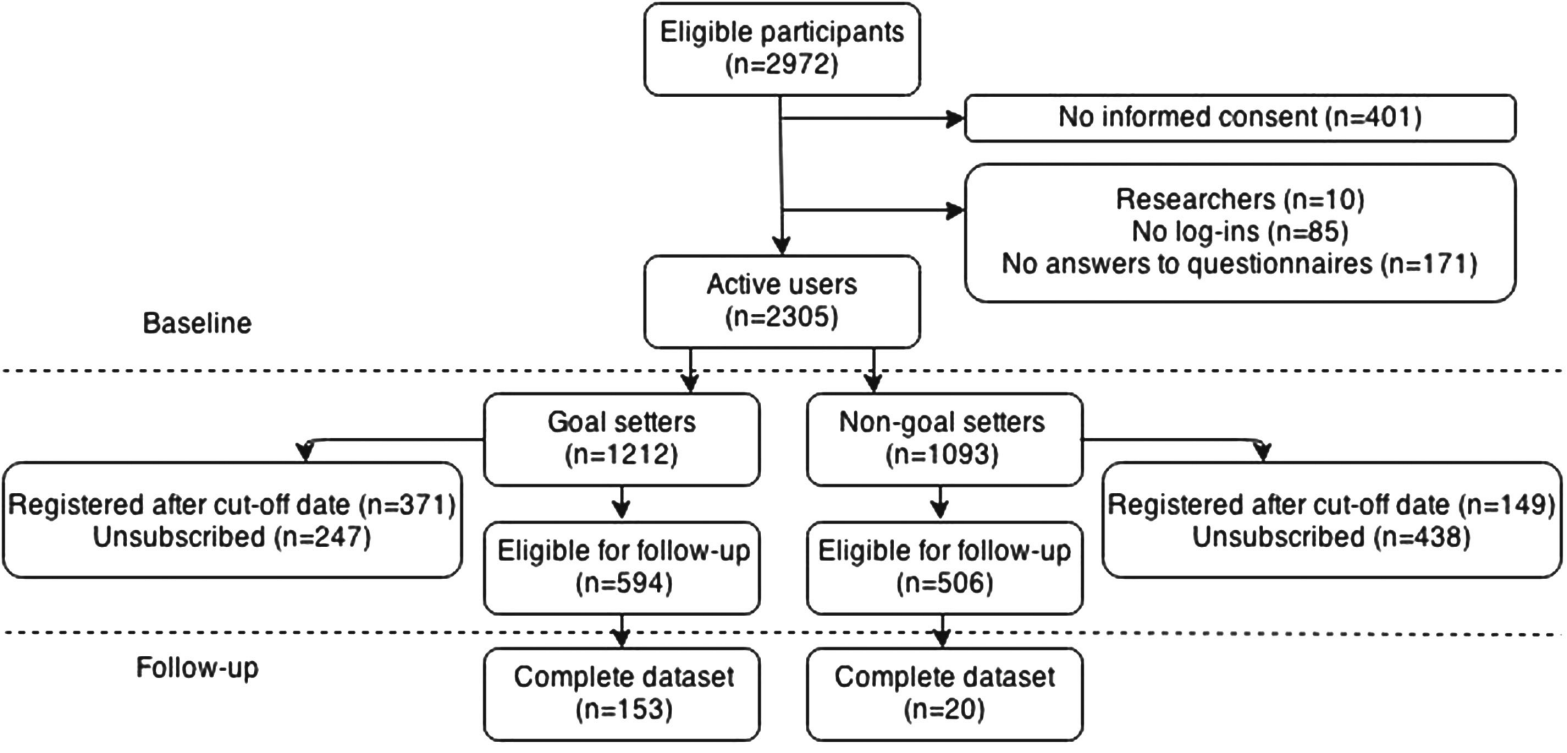
Flowchart of Brain Aging Monitor participants.

A total of 2972 people registered via the website, of whom 2305 became active users (see the flowchart in Figure 1). The mean (SD) age at registration was 51.8 (SD 12.9) years, and 71% of the participants were female. After the baseline measurement, 1212 participants proceeded with setting behavior change goals. Thus, 1093 participants never set a behavior change goal. The participants who set goals were more likely to be female, were less likely to have completed secondary school, and reported less healthy nutrition, and their overall lifestyle score was lower (Table 2).

**Table 2:** Baseline characteristics of the participants who set goals and those who did not.

Characteristic^a^		Goal-setting group (n=1212)	Non–goal-setting group (n=1093)
Age in years, mean (SD)		52.34 (12.21)	51.28 (13.73)
Gender, female, n (%)		862 (71.11)	689 (63.04)^b^
**Education level, n (%)**			
	Secondary school or lower	391 (32.3)	397 (37.2)^c^
	Vocational degree	537 (44.3)	421 (39.5)
	University degree	284 (23.4)	249 (23.3)
Body weight (kg), mean (SD)		75.5 (14.4)	75.6 (14.8)
BMI^d^ (kg/m^2^), mean (SD)		25.2 (4.2)	25.0 (4.4)
**Subjective health, n (%)**			
	Poor	13 (1.1)	16 (1.6)
	Fair	164 (13.5)	144 (13.5)
	Good	796 (65.7)	654 (61.5)
	Very good	187 (15.4)	188 (17.7)
	Excellent	52 (4.3)	61 (5.7)
**Physical activity, n (%)**			
	Inactive	274 (22.6)	206 (19.7)
	Suboptimally active (1-4 days per week)	340 (28.1)	292 (27.9)
	Norm active (≥5 days per week)	598 (49.3)	547 (52.3)
**Exercise, n (%)**			
	Inactive	303 (25.0)	239 (22.9)
	Suboptimally active (1 day per week)	235 (19.4)	210 (20.1)
	Norm active (≥2 days per week)	674 (55.6)	596 (57.0)
Healthy nutrition, range 0-30, mean (SD)		23.6 (4.6)	23.6 (4.8)
Unhealthy nutrition, range 0-14, mean (SD)		5.8 (3.9)	5.4 (3.8)^e^
**Smoking, n (%)**			
	Smoker	85 (7.0)	80 (7.7)
	Ex-smoker	535 (44.1)	455 (44.0)
	Nonsmoker	592 (48.8)	499 (48.3)
**Alcohol, n (%)**			
	Abstainer	257 (21.2)	223 (21.6)
	Drinker (1-5 days per week)	702 (57.9)	571 (55.3)
	Frequent drinker (≥6 days per week)	253 (20.9)	238 (23.1)
**Sleep pattern, n (%)**			
	Poor sleeper	268 (22.2)	208 (20.5)
	Suboptimal sleeper	557 (46.1)	437 (43.1)
	Good sleeper	384 (31.8)	369 (36.4)
Sleep hygiene score, mean (SD)		10.6 (4.0)	10.0 (4.2)
Satisfaction with life, range 5-35, mean (SD)		25.0 (6.3)	25.2 (6.3)
Overall lifestyle score, range 8-24, mean (SD)		17.4 (2.7)	17.7 (2.6)^c^
**Personality questionnaires, mean (SD)**			
	DGSES^f^ (range 10-40)	32.0 (4.3)	32.3 (4.8)
	Positive Affect (range 10-50)	33.9 (5.2)	33.7 (5.5)
	Negative Affect (range 10-50)	21.1 (6.1)	20.4 (5.8)
	Self-Control Scale (range 36-180)	125.6 (13.2)	126.7 (13.1)

^a^ Mean (SD) is presented for continuous and ordinal variables, and n (%) is presented for categorical variables. For healthy nutrition, satisfaction with life, overall lifestyle score, DGSES, Positive Affect, and the Self-Control Scale, a higher score represents better performance; for unhealthy nutrition and Negative Affect, a lower score represents better performance.

^b^*P* ≤.001.

^c^*P*<.05.

^d^ BMI: body mass index.

^e^*P*<.01.

^f^ DGSES: Dutch General Self-Efficacy Scale.

### Use of the Brain Aging Monitor for Goal Setting

The 2305 participants logged on to the BAM a total of 14,225 times. The non–goal-setting group logged on with a mean (SD) of 2.6 (SD 1.9) times per participant, which was significantly fewer than the goal-setting group (9.4, SD 21.3, visits per participant), even when 12 goal-setting participants who logged on >100 times each were excluded (resulting in 7.7, SD 10.0, visits per participant in the goal-setting group; *P*<.001, *r*=.46).

The 1212 goal-setting participants set a total of 2620 lifestyle goals (with 2.2, SD 3.6, goals set per participant). Of these 2620 goals, 2288 were predefined lifestyle goals in the GAS method, and the remaining 332 goals were created without the use of GAS. Of the 2288 prespecified behavior change goals, 1089 (1089/2288, 47.59%) were assessed, and 422 of the completed goals (422/1089, 38.75%) were achieved. Interestingly, all 1089 completed goals (goals that participants set and reported its status on) were completed by a subgroup of 392 participants (392/1212, 32.3% of the goal-setting group); the remaining 820 (820/1212, 67.7%) participants did not complete their goals (so did not report their status) within the study period. Table 3 summarizes the number of goals that the participants set, categorized by lifestyle area; Table 3 also presents statistics for the goal-setting participants who reached their follow-up period and those who did not reach the follow-up period. An interesting result from Table 3 is the notion that goal-setting participants in follow-up had a very high completion rate (575/673, 85.4%) of goals, compared with the goal-setting participants who are not in follow-up (514/1615, 31.8%), but the goal achievement rate was roughly equal: 39.5% (227/575) versus 37.9% (195/514), respectively. Presumably, this is the case because participants who drop out no longer complete their goals and participants who reached their initial goals dropped out of the study, as they no longer perceived added value of the program.

On the reference date for follow-up (March 9, 2014), 1785 participants had been registered for at least 1 year. However, 685 of these participants withdrew from the program, leaving 1100 participants who could provide follow-up data (see Figure 1). In comparison with the 1059 goal-setting participants who were lost to follow-up, the 153 goal-setting participants who reached the follow-up phase had lower BMI (*P*=.03), were more educated (*P*=.04), were more likely to exercise (*P*=.02), and were more likely to be nonsmokers (*P*=.04) at baseline. However, together these differences did not result in a significant difference in overall lifestyle scores between these 2 groups (*P*=.50).

**Table 3:** Overview of goal setting, goal assessment, and goal achievement among the 1212 participants in the goal-setting group.

		Goals set^a^, n	Goals completed^b^, n (%)	Goals achieved^c^, n (%)
**Lifestyle area** ^d^				
	Physical activity	590	270 (45.8)	131 (48.5)
	Weight gain	7	1 (14.3)	0 (0.0)
	Weight loss	516	243 (47.1)	57 (23.5)
	Healthy nutrition	263	126 (47.9)	69 (54.8)
	Unhealthy nutrition	288	143 (49.7)	65 (45.5)
	Smoking	21	6 (28.6)	2 (33.3)
	Alcohol consumption	184	114 (61.2)	47 (41.2)
	Sleep	304	154 (50.7)	39 (25.3)
	Stress	115	32 (27.8)	12 (37.5)
Total number of goals		2288	1089 (47.59)	422 (38.75)
Participants in follow-up^e^				
Total number of goals		673	575 (85.4)	227 (39.5)
Participants not in follow-up^f^				
Total number of goals		1615	514 (31.82)	195 (37.9)

^a^ The number of goals set for each specific lifestyle area.

^b^ Percentage of completed goals out of the number of goals set.

^c^ Percentage of achieved goals out of the number of goals completed.

^d^ The total number of participants in the group is 1212, and the number of participants who completed goals is 392.

^e^ The number of participants who reached follow-up is 153 and, of those, the number of participants who completed goals is 127.

^f^ The number of participants who did not reach follow-up is 1059 and, of those, the number of participants who completed goals is 265.

### Overall and Lifestyle-Specific Lifestyle Changes

After 1 year of BAM intervention, the participants in the goal-setting follow-up group had a significant improvement in their overall lifestyle scores (with a mean change of +0.81, SD 1.92, 95% CI 0.49-1.13; *P*<.001, *d*=0.32). The overall improvement was even greater when only the participants who achieved their goals were taken into consideration (with a mean change of +1.01, SD 1.88, 95% CI 0.61-1.41; *P*<.001, *d*=0.39). Thus, remaining in the program and successfully reaching one’s goals translates to a higher overall lifestyle score over 1 year’s time. Table 4 summarizes the changes in lifestyle factors in the goal-setting group after 1 year in the program. We found that body weight, BMI, physical activity, healthy nutritional habits, unhealthy nutritional habits, hours slept per 24-hour period, and sleep hygiene were positively affected after 1 year’s participation in the BAM. With respect to smoking, no results can be reported, as none of the users in the follow-up group completed smoking-related behavior change goals. With the exceptions of the sleep outcomes and satisfaction with life, the participants who set a goal had significantly lower baseline values in each specific lifestyle area than the participants who did not set goals in that specific lifestyle area. For example, at baseline, the participants who set an exercise goal averaged 1.5 days of exercise per week, whereas the participants who did not set an exercise goal were already exercising an average of 2.3 days per week (*P*=.001).

In addition, using multivariate linear regression analyses, we investigated the association between the total number of goals set by each participant and the change in lifestyle factors from baseline to the 1-year time point; this association was measured for the 173 participants who completed the 1-year follow-up. Our analysis revealed that setting more goals was significantly associated with the participants’ ability to achieve weight loss (adjusted for gender and age at baseline; beta=−.08, 95% CI −.14 to −.02, *P*=.01), reduce BMI (adjusted for gender and age at baseline; beta=−.03, 95% CI −.05 to −.01, *P*=.01), and reduce unhealthy nutritional behavior (beta=−.06, 95% CI −.10 to −.02, *P*=.01). Setting more goals was also positively associated with an improvement in overall lifestyle (beta=.03, 95% CI −.001 to .07, *P*=.06) and healthy nutritional behavior (beta=.05, 95% CI −.001 to .01, *P*=.06). Finally, the total number of goals set was also positively correlated—albeit not significantly—with exercise, alcohol consumption, sleep hygiene, and sleep pattern (data not shown); in contrast, no such trend was found with respect to physical activity or satisfaction with life.

**Table 4:** Change in the lifestyle factors within the goal-setting group (n=153).

Cognitive aging risk factor	Baseline value mean (SD)	Value at year 1 mean (SD)	Mean change (95% CI)^a^	*P* value
Weight (kg)	74.9 (13.5)	74.3 (13.4)	−0.62 (−1.16 to −0.07)	.03
BMI^b^ (kg/m^2^)	24.6 (3.9)	24.4 (3.8)	−0.20 (−0.38 to −0.03)	.03
Physical activity (p/w^c^)	4.1 (2.0)	4.3 (2.0)	+0.27 (0.02-0.52)	.04
Exercise (p/w)	1.9 (1.4)	2.1 (1.4)	+0.17 (−0.02 to 0.37)	.08
Healthy nutrition score	23.7 (3.9)	24.8 (4.0)	+1.09 (0.63-1.56)	<.001
Unhealthy nutrition score	5.7 (3.7)	5.0 (3.7)	−0.67 (−1.05 to −0.29)	.001
Alcohol consumption (p/w)	2.9 (2.4)	2.7 (2.3)	−0.13 (−0.31 to 0.04)	.14
Sleep (hours/24-hour period)	6.8 (0.9)	6.9 (0.9)	+0.12 (0.01-0.22)	.03
Sleep hygiene (points)	10.3 (4.1)	9.0 (4.5)	−1.32 (−1.85 to −0.78)	<.001
Satisfaction with life	24.5 (6.7)	24.2 (7.2)	−0.16 (−1.86 to 0.86)	.75
Overall lifestyle score	17.6 (2.6)	18.4 (2.6)	+0.81 (0.50-1.12)	<.001

^a^ Each mean change and 95% CI was adjusted for gender and age at baseline, and weight and BMI were additionally adjusted for baseline weight.

^b^ BMI: body mass index.

^c^ p/w: per week.

## Discussion

Here, we report that there is a plausible association between utilizing a self-motivated eHealth intervention program for 1 year and one’s overall lifestyle scores by introducing lifestyle-specific health behavior changes that are relevant to cognitive decline. Although the effect size on overall lifestyle score at the individual participant level can be considered moderate (*d*=0.32-0.39) for a therapeutic intervention, from a public health perspective this effect size can be considered highly relevant and may deliver substantial added value to society [33].

### Strengths of the Study

The primary strength of this study is that the intervention can reach and select the participants who score suboptimally in specific lifestyle areas, thereby motivating these specific individuals to set—and reach—realistic lifestyle goals and facilitating long-term health-related changes in behavior. This study therefore demonstrates proof of concept for the BAM intervention, focusing on improving cognitive aging risk factors among employees. Similar studies of other risk factors reported similar effect sizes [39] and concluded that these effects are common among computerized interventions; moreover, evidence suggests that these small to medium effect sizes can translate to large public health gains when implemented on a wide scale [23,24]. Therefore, the need for scalable lifestyle eHealth programs is clear, particularly given that smaller programs—although potentially more effective—lack the cost-effectiveness needed for large-scale public health implementation [40]. This improvement in public health is necessary, because epidemiological studies have found that healthy living—characterized by adherence to multiple healthy behavioral modalities—promotes positive physical and cognitive aging, whereas unhealthy living has a clear negative effect on both [1-3]. Because the BAM is targeted at the working population (a group that is intrinsically self-motivated to stabilize and/or improve their working capacity, particularly in times of economic crisis) it may benefit large populations during this important age window. From a scalability point of view, the funds needed to implement, recruit, and administer the BAM intervention likely favor eHealth over more traditional, expert-led face-to-face interventions [41].

The multimodal nature of cognitive decline justifies choosing a multimodal intervention over single modality programs. Multiple behavior change programs address a broader scope of risk factors, delivering tailored and more comprehensive help to the participant. Moreover, the current public health status in the Netherlands (see Multimedia Appendix 1: Room for improvement in the Netherlands) emphasizes the added value provided by multiple health behavior change interventions. For this reason, we analyzed the effect of the intervention on separate lifestyle factors, as well as the overall lifestyle score. Because one’s overall lifestyle is associated with cognitive function [9,12], measuring changes in overall lifestyle using an aggregated measure seems to make intuitive sense. In support of this approach, other studies measured overall lifestyle in order to create risk profiles for overall mortality, cardiovascular disease, cancer, and diabetes [4,42]. Compared with the relatively small average effect sizes reported by systematic reviews and meta-analyses of other tailored eHealth interventions that focus on more traditional health messages, *d*=0.19 [23], *d*=0.17 [24], and *d*=0.16 [33], our effect size with respect to overall lifestyle change was large (*d*=0.32-0.39). Our participants’ weight loss was similar to previously reported interactive computer-based interventions, particularly given that most weight-loss interventions are aimed at overweight individuals instead of the general population that was targeted in this study [43].

The effectiveness may be explained—at least in part—by the fact that the BAM uses a novel motivation for healthy living (eg, self-monitoring of cognition with games), which by itself has been advocated for many years [44]. However, the general public is generally unfamiliar with the notion that healthy living can affect cognitive health in later life; indeed, to the best of our knowledge, the BAM is the first eHealth intervention that is aimed in this direction. Our results suggest a trend toward a dose-response effect between the number of goals set and lifestyle changes. This is consistent with other studies in which utilization of the intervention predicts outcome measures [21,39]. Consistent with our results, a synthesis of meta-analyses and reviews found that single health behavior change programs were more effective at changing physical activity and dietary behavior, whereas multiple behavior change programs were more effective at inducing weight loss [45]. Given the results of our study, alternative routes to successfully change participants’ physical activity and dietary behavior can be integrated into future eHealth interventions. Thus, although a program may initially be broad in its overall scope, it can subsequently focus on tailored behaviors. A systematic review by Nigg and Long [46] revealed a lack of multiple health behavior change interventions in older adults (ie, aged more than 55 years) for comparison with the effectiveness of single health behavior interventions, underscoring the need for interventions similar to BAM, so that further effectiveness comparisons can be done.

### Limitations of the Study

This study also has some limitations. First, high dropout rates are a well-known limitation in eHealth research in general [21,39,47,48]. Although study retention does not necessarily affect the study’s outcomes [23], low study adherence can hamper external validity and makes results prone to self-selection bias. In this case, the description of this selection bias is also a relevant study result as we meant to identify what subset of the general population would subscribe and adhere to the program and which results could be acquired in that population. There is a form of self-selection bias (eg, higher percentage of female participants) caused by the fact that it is a self-management tool. The BAM could not be forced on participants, but the study outcomes still may point at effectiveness for the type of participants that the intervention program will likely reach in later phases of implementation (ie, external validity). Therefore, it makes the per-protocol results relevant to people who are interested in self-management using eHealth, still resembling the Dutch population of the same age distribution. This in our view increases the external validity. In our study, the average number of times each participant logged on to the BAM is higher than in a similar study [39]; however, the BAM should be improved further in order to increase adherence, thereby optimizing the public health impact. Initial tailoring, repeat notifications, and monitoring are important factors for increasing adherence; however, maintaining the participants’ interest is difficult with eHealth interventions that run longer than a few weeks [21]. Making the program more interactive and increasing the overall attractiveness of the design are two logical steps toward achieving higher adherence rates and increasing societal impact [49,50].

Second, our choice of a quasi-experimental pre-post design was driven by the predicted dropout rate and the setting of the study; this design was the most feasible option for this type of pragmatic field trial [40]. Although a cluster randomized trial would have been preferred, such a design was not feasible given the low number of participating companies. For practical reasons, the companies agreed to include the BAM in their health care policy only if all of their employees would be allocated to the experimental group. Randomizing the participants at each site into control and treatment groups would have posed many practical problems (eg, blinding and allocation criteria [51]). Therefore, given the dropout rate, in an attempt to maximize statistical power by keeping the study group as large as possible, the use of a quasi-experimental design when performing longitudinal self-motivated eHealth research is justified [40]. For the same reason no a priori power analysis and sample size calculation was performed, because it would have been a poorly educated guess at best as we were unaware of the attrition for this innovative Internet-based approach, and because we would need several separate power analyses for the different lifestyle factors addressed. As this was not a randomized controlled trial, we could not follow the CONSORT (Consolidated Standards of Reporting Trials) guidelines at all points to lead us to the feasibility results we were looking for. However, we acknowledge the need for more controlled trials in order to further investigate the causal and dose-response relationship between the intervention uptake and lifestyle change. These trials will all need to find a way to optimally decrease the possible reporting bias that self-reported measures inherently bring in to the study design.

Third, these results are based on self-reported measures, which may have introduced a social desirability bias. It is known that self-reported height, weight, and BMI are reliable using eHealth [52]. The self-management nature and lack of researcher-participant interaction caused multiple participants to state that they “did not feel part of a research project,” decreasing the likelihood of giving socially acceptable answers for the sake of the research team. However, no formal steps were taken to prevent participants from giving socially desirable answers. Moreover, self-reported measures provide a suitable reflection of the way in which participants view their own behavior and therefore serve as a suitable starting point for measuring behavioral changes that are perceived as personally relevant. Our recruitment strategies intentionally favored higher educated, “white collar” participants, as these participants would benefit most from a program with cognitive outcome measurements; such participants are likely to notice a small decline in executive functioning performance at work at a relatively young age. It may also be argued that the overrepresentation of women (71.1% of participants were female) may have affected the outcome of our study. However, this overrepresentation is expected in health-related Internet-based research studies, in which the average female participation rate was 64% [23]; moreover, neither age nor gender was significantly associated with the intervention outcome.

Finally, one could argue that the construction of the overall lifestyle score was—at least to some extent—arbitrary. To the best of our knowledge, no unified method combines—and assigns appropriate weight to—multiple lifestyle outcomes in a way that optimally reflects its effect on cognitive aging. Therefore, we combined 6 lifestyle areas that are known to have an effect on cognitive aging, and we constructed the overall lifestyle score using lifestyle area–specific scores that reflect their respective effects on brain aging. A similar approach has been used previously in other fields [42,53,54]. Combining the self-reported lifestyle into one integer may result in some degree of misclassification and underestimation of the effect on lifestyle. However, in order to create the opportunity for meaningful understanding and also facilitate further analysis between lifestyle and long-term effects (eg, on cognition) we chose to create a single-digit lifestyle score, which can also be used as a starting point to better understand the magnitude of effects of multiple risk factors as well as to facilitate communication on the complete lifestyle effect as a whole.

### Unanswered Questions and Future Research

Asking healthy people in the general population to participate in a lifestyle-improvement trajectory by following a predetermined intervention route that can last for a year (or longer) is notoriously difficult. Our most important recommendation for implementing an eHealth intervention and maximizing program adherence is to design public health programs that are highly flexible, enabling the participants to enter and exit the program freely and at their own convenience while still providing measurements at regular intervals. The ability to measure each participant’s success using a more flexible approach should be addressed in future studies. Methods should be developed to identify pre–follow-up dropouts, who reached a sort of self-aspired end state, as those who successfully completed the intervention. Adapted stepped wedge cluster randomized trial designs may be well suited to this purpose [55]. From a more practical perspective, long-term adherence might be increased by “gamifying” future intervention programs. Adding game components to scientific research might provide the participant commitment and loyalty that many eHealth interventions currently lack [56]. The gamification of eHealth interventions can make the interventions more social, create competition, incorporate a reward system, and enhance motivation. Thus, within the constraints of playing a game, the working mechanisms of current eHealth components can be implemented [57].

### Conclusions

In conclusion, we report adherence to and effectiveness of the BAM program, and we report that this Internet-based, self-motivated and self-managed eHealth intervention, which is aimed at changing multiple health behavior risk factors for cognitive decline, has a positive effect on public health. Given that the participants with the most room for improvement had the greatest change in behavior, and given that the participants who were the most involved with the program had the greatest benefit, we feel that future research with this tool is warranted. More globally, eHealth interventions can achieve more effective and more widespread primary prevention of cognitive decline, thus reducing the predicted strain of aging-related cognitive decline on health care systems in the near future.

## Appendix

Multimedia Appendix 1 Room for improvement in the Netherlands.

Multimedia Appendix 2 Construction of the overall lifestyle score.

## Abbreviations

ANCOVA: analysis of covariance
BAM: Brain Aging Monitor
BAM-COG: Brain Aging Monitor–Cognitive Assessment Battery
BMI: body mass index
GAS: Goal Attainment Scaling
LTMS: long-term monitoring system
SD: standard deviation
