# Supplementary material for: Changing Behavioral Lifestyle Risk Factors Related to Cognitive Decline in Later Life Using a Self-Motivated eHealth Intervention in Dutch Adults
Source: J Med Internet Res. 2016 Jun 17;18(6):e171. doi: 10.2196/jmir.5269 (PMC4930530; doi:10.2196/jmir.5269)
Supplement: Multimedia Appendix 1 [file jmir_v18i6e171_app1.pdf]

Multimedia Appendix 1

In this multimedia appendix the current state of the Dutch population on a number of modifiable lifestyle factors relevant to cognitive aging is described. This is done in order to further clarify the need for eHealth interventions such as the Brain Aging Monitor and its relevance to potentially aid in solving societal challenges.

Room for improvement in the Netherlands

In the Netherlands, the rate of adherence to the Dutch Public Health Physical Activity Guidelines (which calls for a minimum of 30 minutes of moderate physical activity at least 5 days per week) has been stable at approximately 60% of the general population since 2006. However, since 2000, the middle-age population (55-64 years of age) has increased adherence to this guideline. From the year 2000 to 2011, Dutch adults increased the total number of minutes spent daily in physical activity by approximately 19% (from 169 minutes/day to 202 minutes/day); this increased activity primarily reflects increased activity at work, school, and home, but not sports.[1] In contrast, only approximately 50% of elderly individuals (i.e. ≥65 years of age) adhere to the physical activity guidelines. Moreover, in the Dutch workforce, employees reportedly spend 7 hours engaged in sedentary behaviour;[2] this fact alone is cause for alarm, as it reflects a clear negative impact on physical and cognitive health.[3-5]

The average Dutch male consumes 2390-2647 kcal per day, and the average Dutch woman consumes 1849-1956 kcal per day; these values are in accordance with the national guidelines for energy intake. However, only 70% of Dutch adults over the age of 30 eat breakfast on a daily basis. On average, Dutch adults consume 127 grams of vegetables and 97 grams of fruit (and 80 grams of fish on days in which fish is consumed); older women tend to have the healthiest dietary patterns, and younger men tend to have the unhealthiest dietary patterns.[6] Approximately 90%, 84%, and 70-80% of Dutch adults do not meet the nutritional guidelines for consuming vegetables, fruit, and fish, respectively. Finally, it is estimated that 5.2% of total disease burden can be attributed to obesity.[7]

From the year 2000 to 2013, the prevalence of smoking among Dutch adults has declined from 30.6% to 19%, and this decrease can be attributed primarily to several large public health policy changes implemented by the Dutch national government.[8] However, 13.1% of total disease burden can be attributed to smoking cigarettes.[7] Nagelhout and colleagues estimated that from 2011 to 2040, one million Dutch citizens will die of smoking-attributable deaths, and nearly 15% of these deaths might be prevented by introducing an appropriate set of tobacco-controlling laws.[9]

Approximately one in ten Dutch adults between the ages of 40 and 65 are considered heavy drinkers.[10] However, although the prevalence of heavy drinkers has decreased steadily since 2001, more than 80% of Dutch adults between the ages of 40 and 65 drink alcohol, and 2.9-4.5% of the Dutch population suffer from alcohol dependency.[10] As more data emerges supporting the putative ‘J-shaped’ association between alcohol consumption and (for example) cardiovascular health and all-cause mortality becomes available, researchers struggle with the ‘right’ recommendations.[11] In total, 2.8% of the total disease burden may be attributed to alcohol consumption.[7]

Insomnia – although less prevalent in the Netherlands compared to most European countries – is a problem in the Netherlands; the prevalence of insomnia in the Dutch workforce is approximately 14%, with peak prevalence among adults between the ages of 45 and 64.[12]

In the Netherlands, one in three cases of absenteeism from work is caused by work-related stress, making working-related stress the leading occupational disease in this country. One in ten employees

has been absent from work for two consecutive weeks due to work stress-related issues, making these employees more vulnerable to depression and anxiety disorders.[7, 13]

References

1. Hildebrandt VH, Bernaards CM,Stubbe JH, *Trendrapport Bewegen en Gezondheid 2010/2011*. 2013, TNO: Leiden.
2. Jans MP, Proper KI,Hildebrandt VH, Sedentary behavior in Dutch workers: differences between occupations and business sectors. Am J Prev Med 2007. 33(6): 450-4. PMID18022060
3. Lee IM, Shiroma EJ, Lobelo F, Puska P, Blair SN, Katzmarzyk PT, et al., Effect of physical inactivity on major non-communicable diseases worldwide: an analysis of burden of disease and life expectancy. Lancet 2012. 380(9838): 219-29. PMID22818936
4. Owen N, Healy GN, Matthews CE,Dunstan DW, Too much sitting: the population health science of sedentary behavior. Exerc Sport Sci Rev 2010. 38(3): 105-13. PMID20577058
5. Voss MW, Carr LJ, Clark R,Weng T, Revenge of the "sit" II: Does lifestyle impact neuronal and cognitive health through distinct mechanisms associated with sedentary behavior and physical activity? Mental Health and Physical Activity 2014. 7(1): 9-24. PMID16862541
6. van Rossem CTM, Fransen HP, Verkaik-Kloosterman J, Buurma-Rethans EJM,Ocké MC, *Dutch National Food Consumption Survey 2007-2010*. 2011, National Institute for Public Health and the Environment.
7. Hoeymans N, van Loon J, Achterberg P, van den Berg M, Harbers M, den Hartog F, et al., *Volksgezondheid Toekomst Verkenning 2014*. 2014, Rijksinstituut voor Volksgezondheid en Milieu: Bilthoven.
8. Verdurmen J, Monshouwer K, van Laar M,van Bon-Martens M. *Factsheet Continu Onderzoek Rookgewoonten 2013*. 2014; Available from: https://www.trimbos.nl/producten-en-diensten/webwinkel/product/?prod=AF1278;Webcite: http://www.webcitation.org/6cUbKdRIN.
9. Nagelhout GE, Levy DT, Blackman K, Currie L, Clancy L,Willemsen MC, The effect of tobacco control policies on smoking prevalence and smoking-attributable deaths. Findings from the Netherlands SimSmoke Tobacco Control Policy Simulation Model. Addiction 2012. 107(2): 407-16. PMID21906197
10. van Laar MW, Cruts AAN, van Ooyen-Houben MMJ, Meijer RF, Croes EA, Ketelaars APM, et al., *Nationale Drug Monitor Jaarbericht 2012*. 2013, Netherlands Institute of Mental Health and Addiction: Utrecht.
11. Bellavia A, Bottai M, Wolk A,Orsini N, Alcohol consumption and mortality: a dose-response analysis in terms of time. Ann Epidemiol 2014. 24(4): 291-6. PMID24486142
12. Narayanan S, Potthoff P, Guether B, Kanitscheider C,Wiebers IV. *Prevalence of INSOMNIA in Europe, a comparison of Six Countries*. 2009; Available from: http://www.ispor.org/research_pdfs/31/pdffiles/PND1.pdf. Webcite: http://www.webcitation.org/6cUbSRTEm
13. Hupkens C. *Depressiviteit en stress op het werk*. 2004; Available from: http://www.cbs.nl/nl-NL/menu/themas/gezondheid-welzijn/publicaties/artikelen/archief/2004/2004-1383-wm.htm. Webcite: http://www.webcitation.org/6cUdxg28c
