# Supplementary material for: Changing Behavioral Lifestyle Risk Factors Related to Cognitive Decline in Later Life Using a Self-Motivated eHealth Intervention in Dutch Adults
Source: J Med Internet Res. 2016 Jun 17;18(6):e171. doi: 10.2196/jmir.5269 (PMC4930530; doi:10.2196/jmir.5269)
Supplement: Multimedia Appendix 2 [file jmir_v18i6e171_app2.pdf]

Multimedia Appendix 2

Construction of the overall lifestyle score

Lifestyle area	Health Norm Division	Score
Physical Activity	Not active (0-2 days of physical activity per week)	1
	Sub-active (3-4 days of physical activity per week)	2
	Norm active (5-7 days of physical activity per week)	3
Exercise	Not active (0 days of exercise per week)	1
	Suboptimal active (1 day of exercise per week)	2
	Norm active (≥2 days of exercise per week)	3
Healthy Nutritional Behaviour	Lowest tertile of Health Nutrition scores	1
	Middle tertile of Health Nutrition scores	2
	Highest tertile of Health Nutrition scores	3
Unhealthy Nutritional Behaviour	Lowest tertile of Unhealthy Nutrition scores	1
	Middle tertile of Unhealthy Nutrition scores	2
	Highest tertile of Unhealthy Nutrition scores	3
Smoking status	Current smoker	1
	Ex-smoker	2
	Non-smoker	3
Alcohol consumption status[a]	Frequent drinker (≥6 days per week)	1
	Abstainer	2
	Average drinker (≤5 days per week)	3
Sleep status	Poor sleeper	1
	Suboptimal sleeper	2
	Good sleeper	3
Stress status	Lowest tertile of Satisfaction with Life scores	1
	Middle tertile of Satisfaction with Life scores	2
	Highest tertile of Satisfaction with Life scores	3

[a]Please note that moderate consumption is rated over abstaining from alcohol consumption as epidemiological evidence shows that moderate alcohol consumption is beneficially associated with cognitive outcomes in later life. We performed additional frequency checks to make sure that no excessive alcohol intensity was consumed by those who drank moderately. 83.6% of all moderate drinkers did not exceed the healthy limit of 1-2 consumptions per day.
